# *SNHG1* opposes quiescence and promotes docetaxel sensitivity in prostate cancer

**DOI:** 10.1186/s12885-023-11006-x

**Published:** 2023-07-18

**Authors:** Steven P. Zielske, Wei Chen, Kristina G. Ibrahim, Frank C. Cackowski

**Affiliations:** Department of Oncology, Wayne State University and Karmanos Cancer Institute, 4100 John R, MI 48201 Detroit, USA

**Keywords:** Prostate cancer, lncRNA, Cell cycle, Quiescence, *SNHG1*, G_0_

## Abstract

**Background:**

A majority of prostate cancer cells are in a non-proliferating, G_0_ (quiescent) phase of the cell cycle and may lie dormant for years before activation into a proliferative, rapidly progressing, disease phase. Many mechanisms which influence proliferation and quiescence choices remain to be elucidated, including the role of non-coding RNAs. In this study, we investigated the role of a long non-coding RNA (lncRNA), *SNHG1*, on cell proliferation, quiescence, and sensitivity to docetaxel as a potential factor important in prostate cancer biology.

**Methods:**

Publically available, anonymous, clinical data was obtained from cBioPortal for analysis. RNAi and prostate cancer cell lines were utilized to investigate *SNHG1 *in vitro. We measured G_0_ cells, DNA synthesis, and cell cycle distribution by flow cytometry. Western blotting was used to assess G_2_ arrest and apoptosis. These parameters were also investigated following docetaxel treatment.

**Results:**

We discovered that in prostate cancer patients from The Cancer Genome Atlas (TCGA) data set, high *SNHG1* expression in localized tumors correlated with reduced progression-free survival, and in a data set of both primary and metastatic tumors, high *SNHG1* expression was associated with metastatic tumors. *In* vitro analysis of prostate cancer cell lines showed *SNHG1* expression correlated with a quiescent versus proliferative phenotype. Knockdown of *SNHG1* by RNAi in PC3 and C4-2B cells resulted in an accumulation of cells in the G_0_ phase. After knockdown, 60.0% of PC3 cells were in G_0_, while control cultures had 13.2% G_0_. There were reciprocal decreases in G_1_ phase, but little impact on the proportion of cells in S and G_2_/M phases, depending on cell line. DNA synthesis and proliferation were largely halted- decreasing by 75% and 81% in C4-2B and PC3 cells, respectively. When cells were treated with docetaxel, *SNHG1*-depleted C4-2B and PC3 cells were resistant to G_2_ arrest, and displayed reduced apoptosis, as indicated by reduced cyclin B1 and cleaved caspase 3, suggesting *SNHG1* levels may modulate drug response.

**Conclusions:**

Overall, these results indicate *SNHG1* has complex roles in prostate cancer, as it stimulates cell cycle entry and disease progression, but sensitizes cells to docetaxel treatment.

**Supplementary Information:**

The online version contains supplementary material available at 10.1186/s12885-023-11006-x.

## Background

Prostate cancer is characterized by slow progression, and seeding of metastatic sites, most commonly the bone. Clinical dormancy of metastatic prostate cancer may extend for years before reactivation and progression [1]. It is known that prostate cancer tissue contains few proliferating (Ki67^+^) cells [2], which may indicate a significant proportion of cells are quiescent (G_0_ phase). Regulation of quiescence and/or cell cycle (cellular dormancy) likely contributes to clinical dormancy and the response to treatment. However, some cases of prostate cancer proliferate rapidly and follow an aggressive course [3]. The participation of lncRNAs in regulation of cellular quiescence has been little-studied, but is not without precedent. *GAS5* is a lncRNA in which high expression causes pancreatic cancer cells to enter a quiescent state and when expression decreases, cells re-enter a cycling state [4].

*SNHG1* is a 1037 nt lncRNA which was originally described to be upregulated by x-rays in lymphoblastoid cells [5]. Data in prostate cancer has shown *SNHG1* is regulated by dihydrotestosterone treatment, and there is a correlation between high *SNHG1* expression and pathological stage, Gleason score, and time to biochemical recurrence [6]. This indicates a role in prostate cancer, which we expand on here. In neuroblastoma, high *SNHG1* expression is associated with poor survival and is upregulated by MYCN amplification [7]. *SNHG1* has been detected in serum and proposed as a biomarker for hepatocellular carcinoma and lung cancer [8, 9]. In limited studies in non-small cell lung cancer, *SNHG1* knockdown reduced proliferation [10]. *SNHG1* may be part of a feedback loop with p53 through the action of miRNAs derived from the precursor *SNHG1* RNA [11]. *SNHG1* has also been shown to interact with the RNA-binding matrix protein, matrin-3 (*MATR3*) in neuroblastoma [12], and PP2A-c in bladder cancer [13]. The significance of these activities have not been determined. Further information regarding the biological activity of *SNHG1* in cancer is difficult to ascertain, due to the literature being corrupted by fabrication of papers for profit [14, 15]. In fact, the current studies grew from a now retracted paper connecting *SNHG1* to *YAP1* [16]. While those experiments were not reproducible, we discovered *SNHG1* impacted quiescence and chose to pursue those observations to result in the present work.

Cellular quiescence is a so-called “resting” state in which cells are non-dividing, but poised to re-enter the cell cycle when called to do so by external or internal stimuli [17]. Quiescent cells (compared to cycling cells) may be resistant to chemotherapy and radiation due to decreased reactive oxygen species, increased capacity to repair DNA, altered metabolism, differential gene expression, and lack of DNA synthesis/mitosis [17]. Elucidation of quiescent cell mechanisms will aid in targeting this phenotype for cancer treatment, or altering the chemotherapy resistance profile.

The biology of lncRNAs such as *SNHG1* in cancer is an area that requires increased study. As we show here, *SNHG1* is involved in prostate cancer outcome, and can have an outsized effect on cellular quiescence and docetaxel response. High *SNHG1* expression in patient tumors was associated with decreased progression-free survival, compared to low *SNHG1* expression. In prostate cancer cell lines, *SNHG1* silencing inhibited DNA synthesis and arrested cells in G_0_. When treated with docetaxel, *SNHG1*-deficient cells were rescued from G_2_ arrest and apoptosis.

## Methods

### Cell culture

C4-2B (#CRL-3315), DU-145 (#HTB-81), LNCaP (#CRL-1740), and PC3 (#CRL-1435) prostate cancer cells were obtained from American Type Culture Collection (ATCC, Manassas, VA). PNT2 (#95012613) benign prostate cells were obtained from Millipore-Sigma. The use of mVenus-p27K^−^ mutant and mCherry-CDT1 peptide to identify G_1_ and G_0_ phases was first described in mammalian cell culture by Oki et al*.* [18] and then used by us in PC3 cells to form the PC3/VC line [19]. C4-2B/VR cells were constructed by transduction of a p27K^−^-mVenus MMLV vector and a CDT1-RFP709 Lentiviral vector (gift of L. Buttitta) into parental C4-2B cells. All cell lines were grown in RPMI 1640 (#11875135, Gibco, Thermo Fisher Scientific, Waltham, MA) containing 10% fetal bovine serum (#SH3039603HI, HyClone, Cytiva, Marlborough, MA) and 2 mM l-glutamine (#25030164, Gibco, Thermo Fisher Scientific). Cells were grown at 37 °C in a 5% CO_2_ atmosphere. All cell lines have been authenticated and confirmed Mycoplasma negative.

Docetaxel (DTX) was obtained from Cayman Chemical Company (#11637, Ann Arbor, MI) and dissolved in DMSO. For treatment, cells were treated with the specified concentrations of DTX for 2 d, starting the day following siRNA transfection.

In the cell proliferation experiment, daily cell counts were done on an Invitrogen Countess II instrument after initial seeding of 10^4^ cells/well of a 24-well plate.

### EdU assay

EdU (5-ethynyl-2’-deoxyuridine) labeling and staining was carried out using a Click-iT Plus EdU Alexa Fluor 647 Flow Cytometry Kit (#C10634, Invitrogen, Thermo Fisher Scientific). Cells were cultured in 6-well plates at a seeding density of 5 × 10^5^/well for C4-2B, PC3, and DU-145 cells, and 3.6 × 10^5^/well for LNCaP cells. The following day, cells were transfected with siRNA. Two days after transfection, cells were pulse labeled with 10 μM EdU for 2 h, then trypsinized (#25200114, Gibco, Thermo Fisher Scientific), washed with 0.5% BSA in PBS, fixed in 100 μl 0.9% NaCl, 1 ml ice cold methanol, and stored at -20 °C. Following a wash with 0.5% BSA in PBS, the Click-iT EdU reaction was carried out according to the manufacturer’s instructions. Co-staining with propidium iodide was then done as described below and the cells were analyzed by flow cytometry.

### SDS-PAGE and Western blotting

Five hundred thousand cells were seeded into a 6-well plate the day before siRNA transfection. Three days after transfection, and following DTX treatment, cells were lysed inside the wells with RIPA buffer (#89900) containing 1:100 Halt Protease and Phosphatase Inhibitor Cocktail and 1:100 0.5 M EDTA (#784400), and scraping with a cell scraper (reagents all Pierce, Thermo Fisher Scientific). Lysates were sonicated with a probe sonicator and centrifuged at 17,200 × g for 5 min at 4 °C. Protein concentration of supernatants were determined by BCA Assay (#23227, Thermo Fisher Scientific) according to the manufacturer’s instructions.

Samples were prepped for SDS-PAGE by adding 4 × Laemmli Sample Buffer (#161–0747, Bio-Rad, Hercules, CA) containing 10% β-mercaptoethanol to 20 μg of protein and heating at 85 °C for 10 min. Samples were run on a 4**-**15% Mini-PROTEAN TGX Stain-Free precast gel (#4568083, Bio-Rad) and transferred to PVDF. Membrane was blocked in EveryBlot Blocking Buffer (#12010020, Bio-Rad) for 20 min., then incubated in primary antibody diluted in EveryBlot overnight at 4 °C. Blot was then washed 3 × in Tris-buffered saline, pH 7.4, 0.05% Tween-20 (TBS/T). Blot was incubated in secondary antibody diluted in EveryBlot for 1 h at room temperature and washed 3 × in TBS/T. Proteins were visualized with Supersignal West Pico PLUS Chemiluminescent substrate (#34580, Thermo Fisher Scientific). Some blots were stripped with Restore Western Blot Stripping Buffer (#21059, Thermo Fisher Scientific) and reprobed with additional antibodies following blocking, as above. Images were taken with a Bio-Rad ChemiDoc Touch Gel Imaging System and quantified with Bio-Rad Image Lab 6.1 software.

Antibodies used in these studies are as follows: anti-β-actin (clone 13E5, #4970S), anti-caspase 3 (clone 3G2, #9668S), anti-PARP (#9542S), all rabbit monoclonals, and anti-cyclin B1 (clone V152, #4135S), a mouse monoclonal (all from Cell Signaling Technology, Danvers, MA). Anti-β-actin and anti-cyclin B1 antibodies were used at 1:2000 dilution, other primary antibodies at 1:1000. Secondary antibodies were anti-mouse IgG-HRP (#7076S) used at 1:2000 dilution, and anti-rabbit IgG1-HRP (#7074S) used at 1:3000 dilution (Cell Signaling Technology).

### Flow cytometry

Flow cytometry was done at the Microscopy, Imaging and Cytometry Resources Core at Wayne State University, School of Medicine. EdU-Alexa Fluor 647 × PI dual stained cells, PI-only stained cells for cell cycle analysis, and C4-2B/VR cells were analyzed on a Cytek Biosciences Northern Lights instrument. PC3/VC cells were analyzed on a Becton-Dickinson LSR II instrument. Compensation controls consisted of parental PC3 cells labeled with anti-human HLA-A, B, C-FITC (#311404) and anti-human HLA-A, B, C-PE/Dazzle 594 (#311440) antibodies (Biolegend). All viable cell analyses included the addition of dapi for gating out dead cells. Data was analyzed using FlowJo v10.8.0 software (FlowJo, Ashland, OR). Final dot plots or histograms were obtained after gating on the main population from side scatter-forward scatter, plus singlets, minus dead cells.

PC3/VC cells from a confluent plate were split 1/5 to achieve a low density. The next day, media was replaced with serum-free OptiMEM (#31985070, Gibco, Thermo Fisher Scientific). Then the following day, cells were trypsinized, washed in PBS containing 2% FBS, and sorted on a Sony SH800 Cell Sorter into complete RPMI media. Cells were then centrifuged, and Qiagen RNeasy lysis buffer was added to the cell pellet for subsequent RNA isolation.

### RT-qPCR

RNA was isolated from cells using an RNeasy Mini-Prep kit (#74106, Qiagen, Germantown, MD). RNA was reverse transcribed into cDNA using a SuperScript IV First Strand Synthesis System (#18090010, Invitrogen, Thermo Fisher Scientific). Quantitative PCR (qPCR) was done using a TaqMan Gene Expression Master Mix (#4369016, Applied Biosystems, Thermo Fisher Scientific) and the following TaqMan Gene Expression Assays: *SNHG1* (Hs00411543), *ACTB* (Hs99999903_m1), and *GAPDH* (Hs99999905_m1). *SNHG1* expression was determined relative to the housekeeping genes *ACTB* and *GAPDH*. PCR was performed on a Bio-Rad CFX Connect Real Time PCR machine.

### RNAi

Cells were seeded into 24-well (10^5^ cells) or 6-well (5 × 10^5^ cells) the day before siRNA transfection. The day of transfection, media was replaced with OptiMEM and siRNA was combined with Lipofectamine RNAiMAX (#13778150, Invitrogen, Thermo Fisher Scientific) and added to each well according to the manufacturer’s instructions. If cells were not harvested the next day, the OptiMEM was replaced with complete RPMI for the duration of the experiment.

### Cell cycle

We seeded 2.5 × 10^5^ C4-2B, DU-145, or PC3 cells, or 5 × 10^5^ LNCaP cells, into 6-well plates the day before siRNA transfection. The day after transfection, media was replaced with complete RPMI. Cells were treated with 0 or 20 nM DTX for 2 d as described above. Then cells were trypsinized and washed with PBS containing 0.5% BSA. Cells were centrifuged and pellets resuspended in 100 μl cold 0.9% NaCl. Then, 1 ml ice cold 100% methanol was added to fix the cells. The day of flow cytometry, cells were washed with 0.5% BSA, PBS and resuspended in FxCycle PI/RNase Staining Solution according to the manufacturer’s instructions (#F10797, Invitrogen, Thermo Fisher Scientific). Data was analyzed using FlowJo software to determine the proportion of apoptotic cells, and the proportion in G_1_, S, and G_2_/M phases using the cell cycle tool and Watson model within the software.

For discrimination of G_0_ cells from other phases, live cell flow cytometry on PC3/VC or C4-2B/VR cells was performed, with dual positive (p27K^−^-mVenus x CDT1-mCherry or p27K^−^-mVenus x CDT1-RFP709) cells indicating G_0_ phase, single positive CDT1-mCherry or CDT1-RFP709 indicating G_1_, and dual negative cells comprised of S, G_2_, and M phases.

### Docetaxel IC_50 _determinations

We plated 5,000 cells of each cell line (C4-2B, PC3, LNCaP, 22Rv1, and DU-145) into wells of a 96-well plate. The day after plating, we added 0, 0.2, 0.5, 1, 2, 5, 10, or 20 μM docetaxel to quadruplicate wells. Cell were incubated 5 days and proliferation was determined using a CellTiter 96® AQueous One Solution Cell Proliferation Assay (#G3580, Promega, Madison, Wisconsin). Data was graphed in Graphpad Prism using an inhibitor versus response (3 parameters) model, which determined IC_50_ values.

### Clinical data analysis

The TCGA Prostate Adenocarcinoma cohort with 494 patients (PanCancer Atlas) was utilized to assess the relationship of SNHG1 expression and patient outcomes. The mRNA expression was batch normalized from illumine HiSeq_RNASeqV2 prior to release on cBioPortal. *SNHG1* expression was stratified into two groups, high and low, at the median of expression values. Patient progression free survival (PFS) was estimated with the Kaplan-Meier method. Death without tumor progression was censored at time of death. As a total of four death events out of 494 patients (< 1%) occurred without tumor progression, competing risk analysis was not performed. Relationship between the *SNHG1* high/low expression and PFS was modeled with Cox multivariable regression, adjusted for age, stage, and tumor burden. Other clinical variables such as race had 68% missing values, which will not generate meaningful statistics, and were not included in the model. R (version 4.1.0) was used for statistical analysis.

### Statistical analysis

Statistical analysis for non-clinical data was done using Graphpad Prism (v8.4.2) software (Graphpad Software, LLC, San Diego, CA). Paired or unpaired Student t test (as appropriate) was used as indicated in Figure legends. An exponential growth equation was used to fit cell count data. Some clinical data was analyzed using a Chi-squared test, or unpaired Student t test, as indicated. A Deming model II linear regression was used to fit data in Fig. 8.

## Results

### Clinical correlates of *SNHG1* expression

**Fig. 1:**
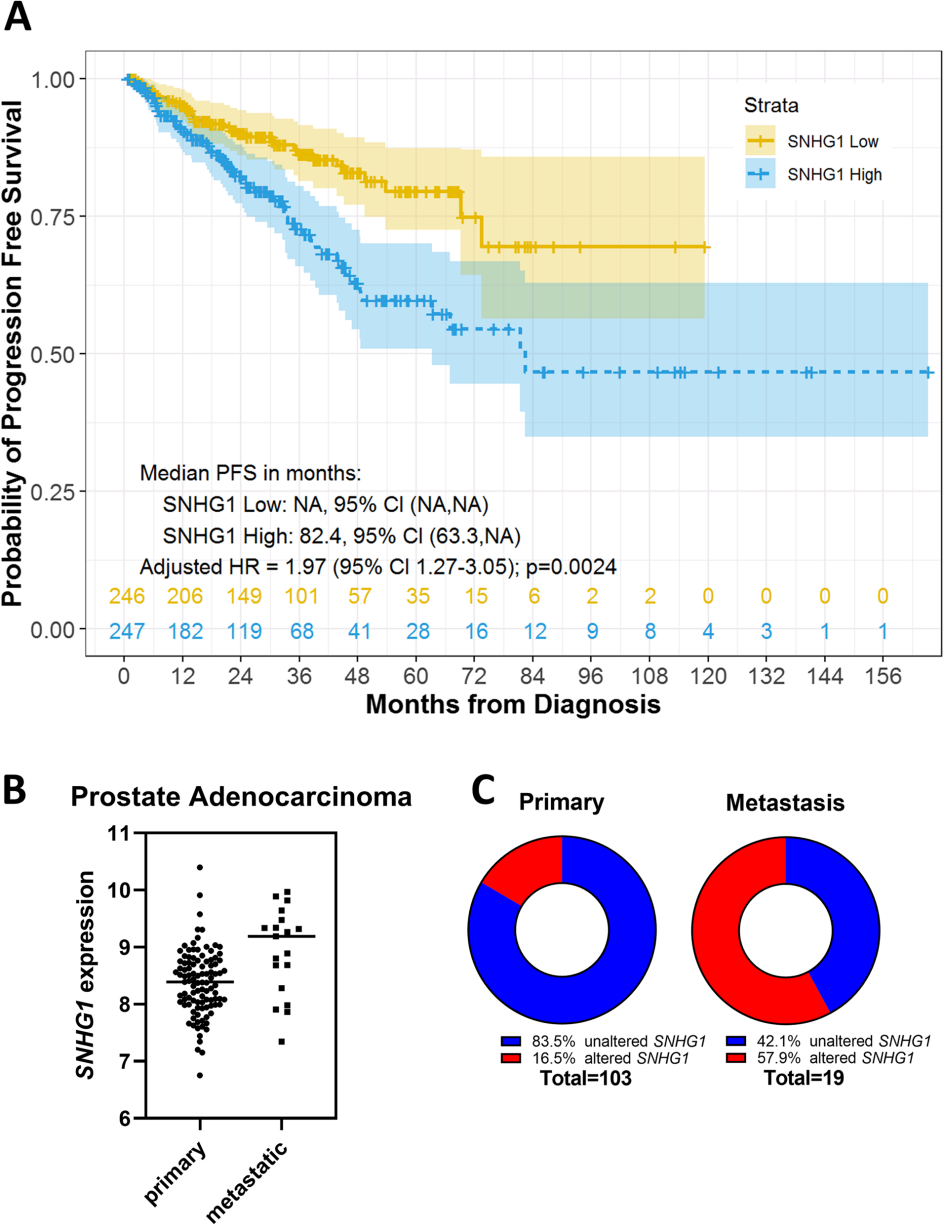
High *SNHG1* expression correlates with reduced PFS and a metastatic phenotype. **A** PFS stratified by *SNHG1* high/low expression in the TCGA prostate adenocarcinoma PanCancer Atlas data set. Median PFS was estimated with Kaplan-Meier method. Adjusted HR was estimated with the multivariable Cox model adjusted for age, stage, and tumor burden. **B** *SNHG1* expression in primary and metastatic tumors from patients in the MSKCC data set. Line is mean, *N* = 122. Statistical analysis by Student’s t test: *P* = 0.0003. **C** Comparison of the number of patients with ‘altered’ *SNHG1* expression in primary and metastatic tumors, from the MSKCC data set. Statistical analysis by Chi-squared test: *P* = 0.0003

We utilized publically available clinical data with no personally identifiable information (*N* = 494) from cBioPortal [20, 21] to investigate *SNHG1* association with clinical features of prostate cancer. When we compared PFS in the TCGA dataset (*N* = 493, due to one patient missing *SNHG1*) [22] between the low and high *SNHG1*-expressing groups, we found that *SNHG1* was an independent prognostic factor for PFS, adjusted for age at diagnosis, stage, and tumor burden, with an adjusted hazard ratio (HR) of 1.97 (*P* = 0.0024; Fig. 1A).

We next compared primary versus metastatic tumors from 126 patients in the MSKCC dataset [23]. Metastatic tumors showed higher *SNHG1* expression than primary tumors (*P* = 0.0003; Fig. 1B). Among the 22% of samples with altered *SNHG1* levels in the dataset, all but one showed *SNHG1* overexpression. The proportion of altered (over- or under-expression) *SNHG1* in primary versus metastatic tumors was also analyzed. In primary tumors, 17% showed altered *SNHG1*, however, in metastatic tumors, the proportion of altered *SNHG1* was 58% (*P* = 0.0003; Fig. 1C).

These data show that *SNHG1* expression plays a role in prostate cancer progression and overexpression associates negatively with outcome. In addition, *SNHG1* overexpression is more commonly associated with metastatic tumors, suggesting a contribution to metastasis biology.

### Effect of *SNHG1* on proliferation

**Fig. 2:**
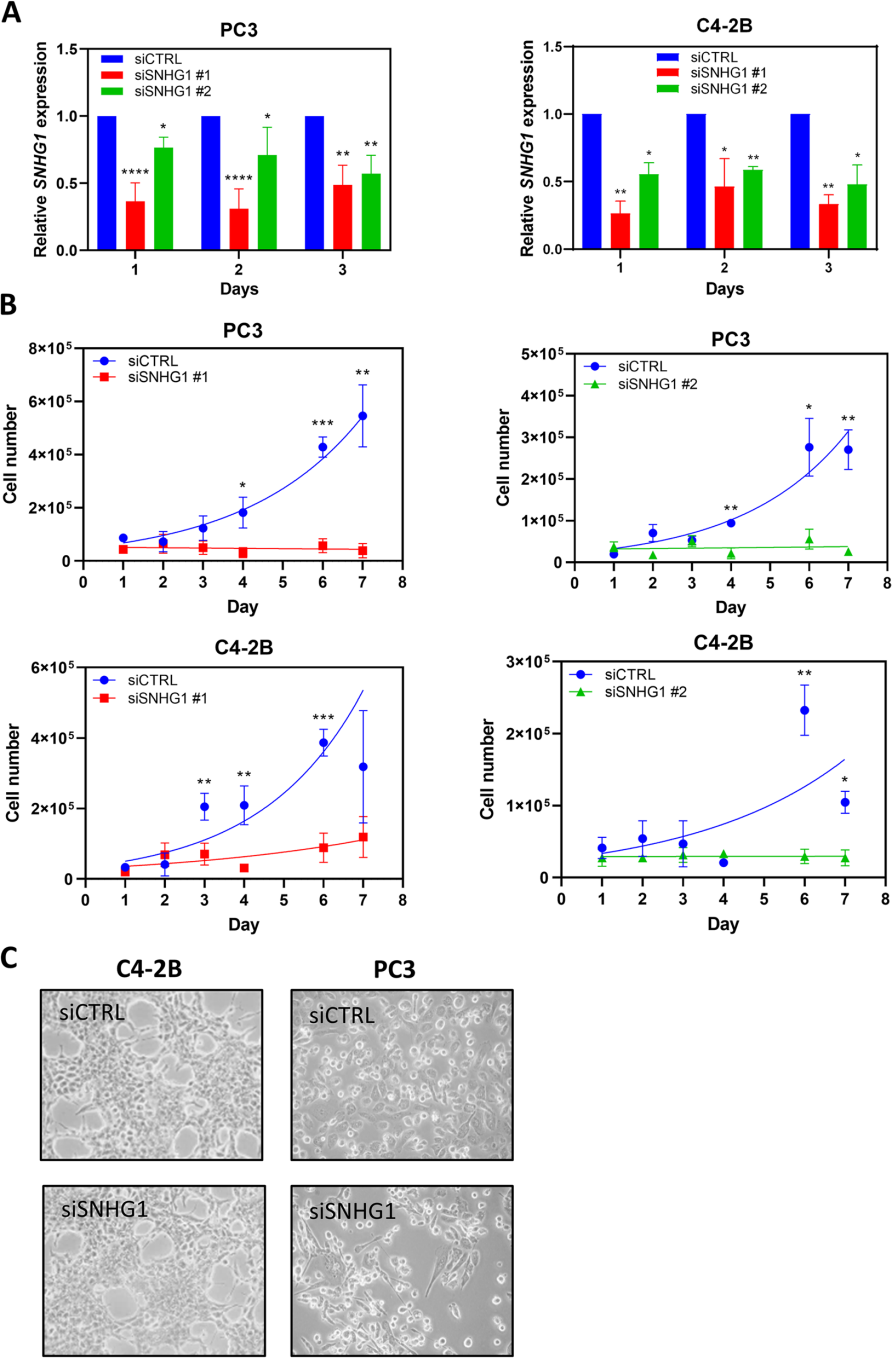
*SNHG1* knockdown suppresses cell proliferation. **A** Using RT-qPCR, *SNHG1* expression was assessed for 3 d following transfection with siRNAs #1 and #2 targeting *SNHG1* in PC3 and C4-2B cells, relative to control siRNA-transfected cells.** B** PC3 and C4-2B cells were transfected with siCTRL, siSNHG1 #1, or siSNHG1 #2 siRNA, and counted for up to 7 d. Data was fit to an exponential growth curve. **C** Photographs of C4-2B and PC3 cells 2d after *SNHG1* knockdown. All data points represent mean ± SD with *N* = 3–6. All statistical analysis done using Student’s t test: *, *P* < 0.05; **, *P* < 0.01; ***, *P* < 0.001; ****, *P* < 0.0001

A limited study of non-small cell lung cancer showed that *SNHG1* knockdown resulted in reduced proliferation [10]. To assess the impact of *SNHG1* on proliferation in prostate cancer cells, we performed *SNHG1* RNAi in *p53* (*TP53*) null PC3 cells, and *p53* wild-type C4-2B cells and measured cell counts over 7 d. Wild type and null p53 status cells were chosen because of the effect of p53 on cancer cell biology and cell cycle, and we wanted to consider the possibility that knockdown of *SNHG1* might have differential effects based on p53 status. We used two different *SNHG1* siRNAs to achieve knockdown in PC3 cells of 51**-**64% and 24**-**43% using siSNHG1 #1 and siSNHG1 #2, respectively. In C4-2B cells, we achieved 54**-**74% and 41**-**52% knockdown using siSNHG1 #1 and siSNHG1 #2, respectively (Fig. 2A). A modest knockdown of 32% continued at 7 d after transfection (not shown). PC3 cells undergoing *SNHG1* RNAi ceased to proliferate, or had a doubling time of 25 d, using siSNHG1 #1 or siSNHG1 #2 siRNAs, respectively, while respective control cells had a doubling time of 2.0 d and 1.8 d (Fig. 2B). Likewise, control C4-2B cells had a doubling time of 1.8 d or 2.6 d, but after *SNHG1* knockdown, cell proliferation slowed and the doubling time increased to 3.7 d and 279 d, using siSNHG1 #1 and siSNHG1 #2, respectively (Fig. 2B).

Analysis of cell morphology after *SNHG1* knockdown revealed no difference between siCTRL and siSNHG1 transfected C4-2B and PC3 cells (Fig. 2C). There were no obvious indications of toxicity, change in epithelial phenotype, attachment, granularity, or changes associated with senescence. A senescence-associated β-galactosidase activity assay was negative for senescence (not shown).

**Fig. 3:**
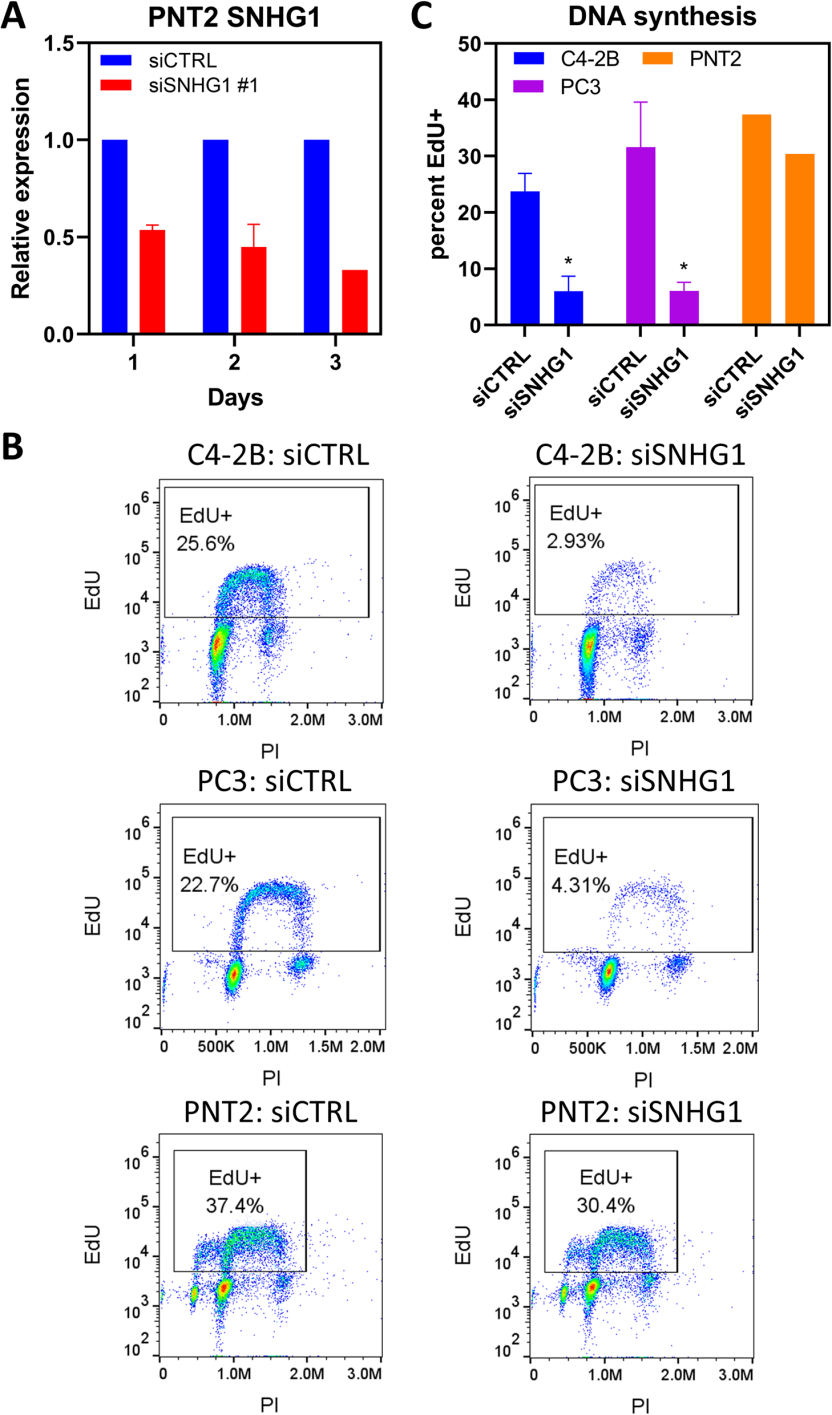
SNHG1 knockdown reduces DNA synthesis in cancer cells. **A** RNAi efficiency of siSNHG1 #1 siRNA was assessed in PNT2 cells by RT-qPCR. **B** Representative flow cytometry density plots of C4-2B, PC3, and PNT2 cells analyzed by flow cytometry following transfection with siCTRL or siSNHG1 siRNA, and a pulse with EdU to determine ongoing DNA synthesis. **C** Quantitation of cells labeled with EdU and analyzed by flow cytometry for DNA synthesis. C4-2B and PC3 data represent mean ± SD. *N* = 3. Statistical analysis done using Student’s t test: *, *P* < 0.05. PNT2 data represent *N* = 1–2

Since proliferation was reduced or eliminated after *SNHG1* silencing, we next investigated whether DNA synthesis was affected in C4-2B and PC3 cells, and the benign prostate cell line, PNT2. Analysis of DNA synthesis by EdU labeling 2 d after siRNA transfection showed a substantial decrease in the proportion of EdU-labeled cells in both cancer cell lines (Fig. 3B). C4-2B transfected with siCTRL had an average of 24% EdU^+^ cells while silencing of *SNHG1* resulted in 6.0% EdU^+^ cells (*P* < 0.03; Fig. 3C). Silencing *SNHG1* in PC3 cells caused the proportion of EdU^+^ cells to go from 32% to 6.1% (*P* < 0.03; Fig. 3C). Equivalent results were obtained with DU-145 and LNCaP cells (Additional file 1). We then investigated DNA synthesis in PNT2 cells. *SNHG1* knockdown efficiency of 46**-**67% was obtained (Fig. 3A). We found there was little effect on DNA synthesis following *SNHG1* knockdown, with controls cells having 37% EdU^+^ cells and SNHG1-deficient cells having 30% EdU^+^ cells (Fig. 3B, C).

These data show that knockdown of *SNHG1* halted, or nearly halted, prostate cancer proliferation independent of p53. DNA synthesis was dramatically reduced under conditions of incomplete *SNHG1* knockdown. The effect was not observed in benign prostate cells.

### *SNHG1* RNAi induces quiescence

**Fig. 4:**
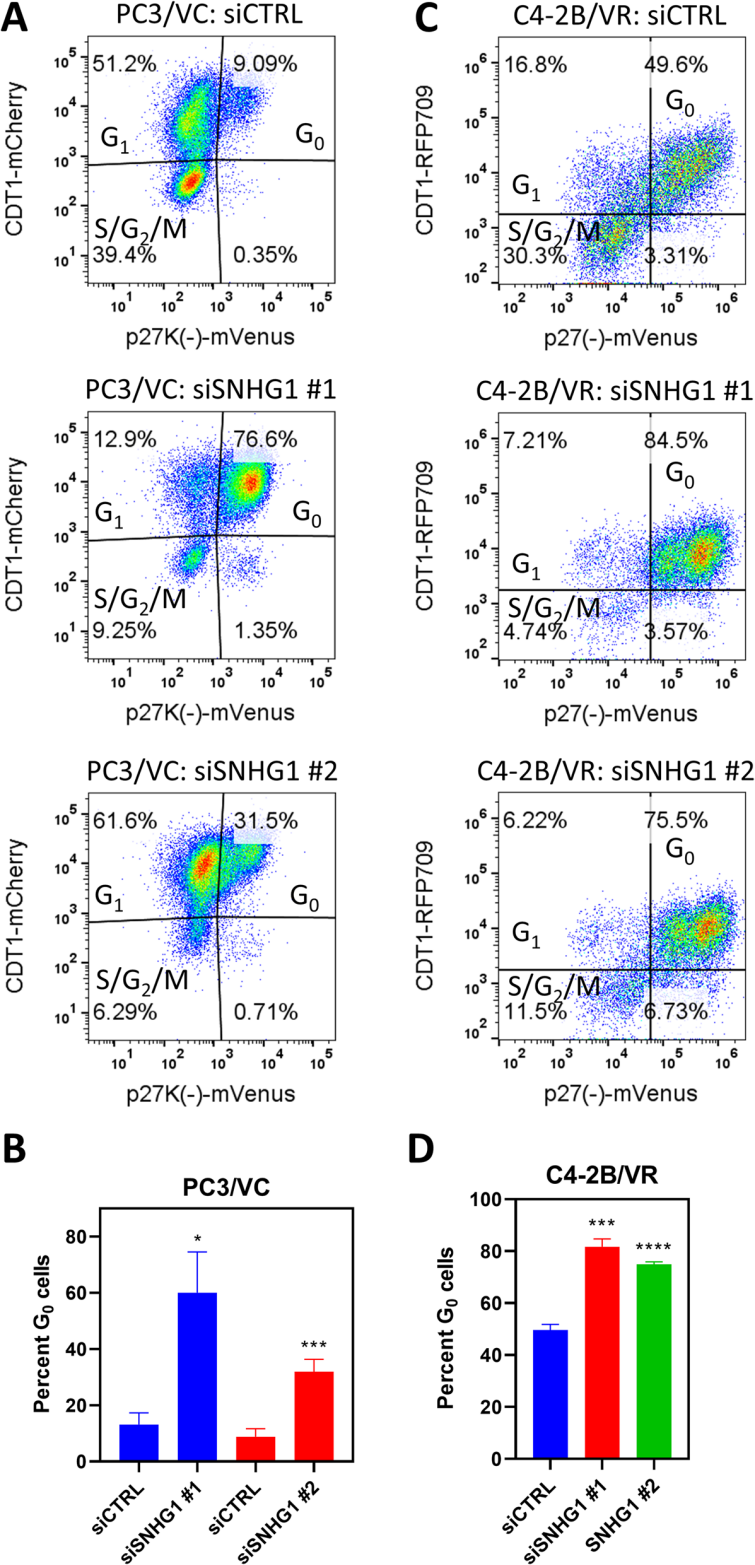
*SNHG1*-deficient cells accumulate in G_0_ phase. **A** Representative flow cytometry density plots of PC3/VC cells following SNHG1 knockdown, or not (siCTRL), with two different siRNAs, siSNHG1 #1 and #2. **B** The proportion of G_0_ cells in siCTRL, siSNHG1 #1, or siSNHG1 #2-transfected PC3/VC cells. **C** Representative flow cytometry histograms of C4-2B/VR cells following SNHG1 knockdown with siSNHG1 #1 and #2 siRNA. **D** The proportion of G_0_ cells in siCTRL, siSNHG1 #1, or siSNHG1 #2-transfected C4-2B/VR cells. All graphed data represent mean ± SD, *N* = 3-6. All statistical analysis done using Student t test: *, *P* < 0.05; **, *P* < 0.01; ***, *P* < 0.001; ****, *P* < 0.0001

Since cell proliferation and DNA synthesis were reduced, we investigated whether cells had shifted into a quiescent, G_0_ state. To accomplish this, we utilized PC3/VC cells containing fluorescent protein markers mVenus and mCherry to indicate endogenous p27 (CDKN1B) and CDT1 (FUCCI cell system) expression, respectively, and C4-2B/VR cells containing mVenus and RFP709 to indicate p27 and CDT1 expression, respectively [18, 23]. CDT1 is expressed in G_1_ and G_0_ cells, while p27 is expressed in G_0_ cells. In PC3/VC cells undergoing *SNHG1* silencing, there was a large shift of cells into G_0_ phase, from as few as 9.09% in control cells to 76.6% following *SNHG1* knockdown with siSNHG1 #1 siRNA (Fig. 4A). Using a second siRNA (siSNHG1 #2), the percentage of G_0_ cells increased to 31.5%, indicating the effect was specific (Fig. 4A). The smaller increase in G_0_ cells was likely due to the reduced knockdown efficiency of siSNHG1 #2 (Fig. 2A). The average percentage of G_0_ in cells transfected with siSNHG1 #1 was 60.0%, while control cells had an average of 13.2% in the G_0_ phase, a 5.1-fold increase (*P* = 0.048; Fig. 4B). Using siSNHG1 #2, we similarly found the average proportion of G_0_ cells to increase from 8.8% in siCTRL to 31.9% in siSNHG1 #2, a 3.6-fold increase (*P* < 0001; Fig. 4B). In C4-2B/VR cells, *SNHG1* silencing caused an increase in G_0_ cells from an average 49.6% in siCTRL cells to 81.7% and 75.0% in cells transfected with siSNHG1 #1 and siSNHG1 #2, respectively (*P* < 0.001, Fig. 4C, D).

While a significant proportion of *SNHG1* depleted cells were found to be in G_0_ phase, the lack of DNA synthesis and cell growth suggested there may be effects on other cell cycle phases, or an increase in apoptosis. We analyzed apoptosis and cell cycle using PI staining and flow cytometry. We found the cell cycle distribution (G_0_/G_1_, S, or G_2_/M) between control and *SNHG1* knockdown cells to be the same in C4-2B cells (Fig. 6A, B). Apoptosis was 1.0% or less in control cells, with no significant difference following *SNHG1* knockdown (Fig. 6B). Similar data were obtained with DU-145 and LNCaP cells (Additional file 2). LNCaP and DU-145 cells showed a small decrease in S and G_2_/M phase cells following knockdown that, in some cases, reached statistical significance (Additional file 2). We could not quantify cell cycle distribution by PI in PC3 cells due to excessive polyploidy. However, histogram traces seemed to indicate some reduction in G_2_ cells after *SNHG1* silencing (Fig. 6A).

**Fig. 5:**
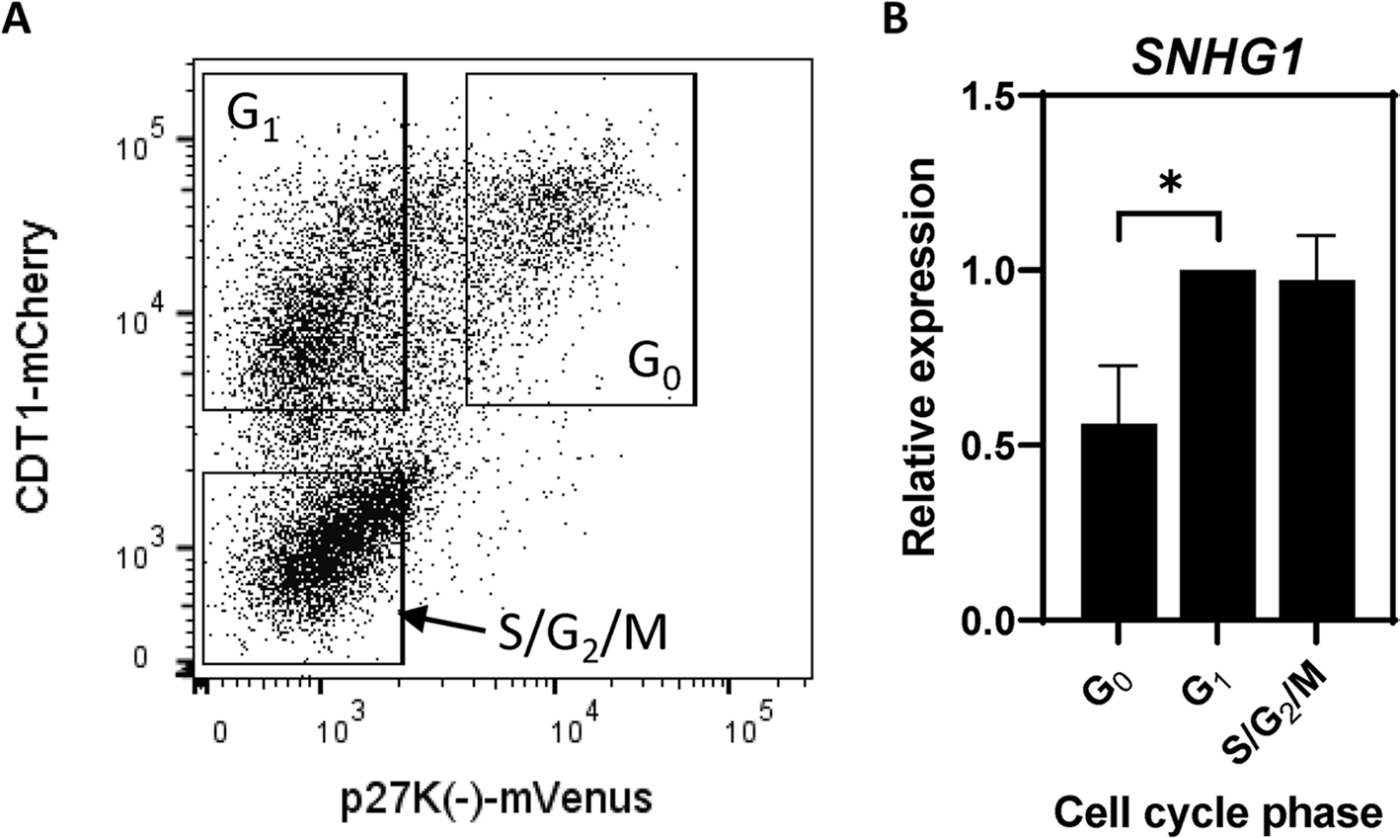
Quiescent cells express low *SNHG1*. PC3/VC cells were FACS sorted into G_0_, G_1_, and S/G_2_/M populations. **A** Representative flow cytometry dot plot showing the gates used in sorting populations for subsequent RT-qPCR. **B** Sorted cells were subjected to RT-qPCR to quantify *SNHG1* levels. Data represent mean ± SD, *N* = 4. Statistical analysis done using Student t test: *, *P* < 0.05

Since knockdown of *SNHG1* led to accumulation of cells in G_0_, we postulated that *SNHG1* expression may be lower in quiescent cells, and higher in cycling cells under normal culture conditions. To investigate this, we plated PC3/VC cells for 2 d and then FACS-sorted them based on the p27K^−^-mVenus and CDT1-mCherry markers into G_0_, G_1_, and S/G_2_/M populations (Fig. 5A). We then quantified *SNHG1* levels by RT-qPCR and found that the G_0_ population expressed 44% lower levels of *SNHG1* than the G_1_ population (*P* = 0.031, Fig. 5B). Expression in the S/G_2_/M population was similar to G_1_, with 3% lower expression.

Overall, these data show that *SNHG1* expression is associated with a proliferation phenotype and that loss of *SNHG1* results in accumulation of cells in a quiescent, G_0_ state. Knockdown of *SNHG1* in multiple prostate cancer cell lines prevented cells from exiting G_0_, or suppressed cycling cells such that they entered G_0_, as determined by expression of the quiescent cell marker p27. Cell cycle analysis showed small disruptions in S and G_2_/M populations according to DNA content, following *SNHG1* knockdown.

### Effect of *SNHG1* on cell cycle and docetaxel response

Docetaxel is used to treat metastatic prostate cancer and exerts its activity through stabilization of microtubules and induction of BCL-2 phosphorylation, resulting in G_2_/M arrest, mitotic catastrophe, and apoptosis [24]. *SNHG1* expression is associated with cell cycle progression. We thus investigated the impact of *SNHG1* on cell cycle and apoptosis responses to docetaxel treatment in vitro.

**Fig. 6:**
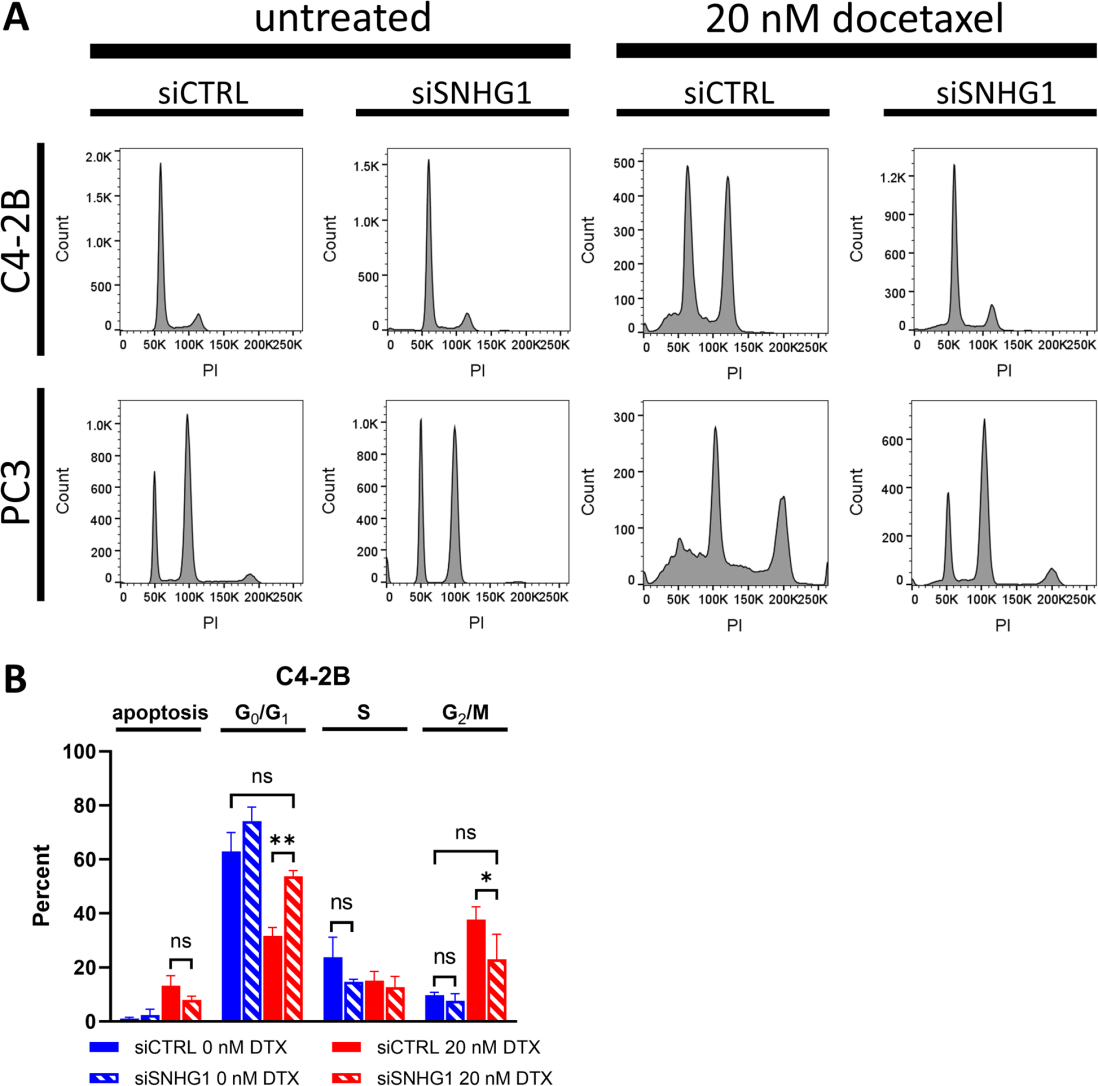
*SNHG1* silencing is protective against DTX. We treated C4-2B and PC3 cells with DTX and analyzed changes in cell cycle and apoptosis. **A** Representative histograms of PI-stained cells with or without *SNHG1* knockdown and with or without treatment with 20 nM DTX. **B** Results of quantitation of apoptotic, G_0_/G_1_, S, and G_2_/M populations in untreated or DTX treated, *SNHG1* silenced or not, C4-2B cells. Data represent mean ± SD, *N* = 3-4. Statistical analysis was done using Student’s t test: ns, not significant; *, *P* < 0.05; **, *P* < 0.01

Treatment of C4-2B and PC3 cells with 20 nM docetaxel for 2 d led to a G_2_ arrest and an increase in apoptosis (Fig. 6A). However, when *SNHG1* was silenced by RNAi, the G_2_ arrest was significantly decreased, and apoptosis showed a trend toward reduction. The increase in G_0_/G_1_ in treated *SNHG1*-knockdown cells compared to treated control cells was significant, as was the reduction in the G_2_/M population (Fig. 6B), indicating that knockdown of *SNHG1* abrogated the docetaxel-mediated G_2_ arrest. We quantified cell cycle phase in C4-2B, but not PC3 cells because PC3 cells contain a significant number of multi-nucleated and polyploid cells, confounding cell cycle quantitation by DNA content. However, similar results were obtained in LNCaP and DU-145 cells (Additional file 2). In some cases, the docetaxel-induced G_2_/M arrest was almost completely negated. After docetaxel treatment, the proportion of G_2_/M cells in siCTRL-transfected C4-2B increased from 9.7% to 37.7% (*P* < 0.03; Fig. 6B). Silencing of *SNHG1*, however, abrogated this increase, with 23.0% in C4-2B cells (*P* < 0.05, compared to treated siCTRL; Fig. 6B). PC3 cells also clearly showed a reduction in the size of the 8N G_2_ peak. Changes in the G_2_/M population were reflected in changes in the G_0_/G_1_ population. The proportion of G_0_/G_1_ cells in untreated siCTRL, treated siCTRL, and treated siSNHG1 C4-2B was 63.0%, 31.7%, and 53.8%, respectively, indicating a near return to normal levels of G_0_/G_1_ after *SNHG1* silencing (Fig. 6B). Similar results were obtained in DU-145 and LNCaP cells (Additional file 2). Apoptosis was reduced in treated siSNHG1 cells, compared to treated siCTRL, however, the decrease was not statistically significant in this assay. PC3 cells show what appears to be a large reduction in apparent apoptotic cells according to the PI histograms.

**Fig. 7:**
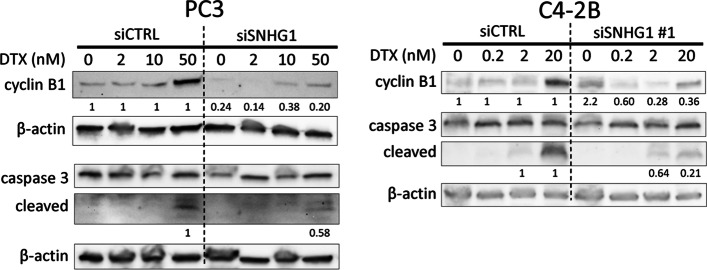
*SNHG1* silencing results in reduced G_2_ phase and apoptosis markers after DTX treatment. Representative Western blots showing the apoptosis marker, cleaved caspase 3, and the G_2_ marker, cyclin B1. β-actin is loading control. For PC3, two separate blots are shown. For C4-2B, a single blot was probed for multiple proteins. Numbers indicate band density versus siCTRL, normalized to β-actin. Full length blots are presented in Additional file 3

We then performed Western blots on PC3 and C4-2B cells to further analyze G_2_ phase effects and apoptosis due to docetaxel. Expression of the G_2_ marker, cyclin B1, increased with increasing dose of docetaxel in siCTRL for both cell lines, signifying a G_2_ arrest in response to drug (Fig. 7). After *SNHG1* knockdown, the level of cyclin B1 decreased, with 0.20 and 0.36 as much as siCTRL in 50 nM docetaxel treated PC3 and 20 nM docetaxel treated C4-2B, respectively. Apoptosis was reduced in treated cells undergoing *SNHG1* silencing (Fig. 7). Caspase 3 cleavage became evident at 50 nM and 20 nM docetaxel in siCTRL PC3 and C4-2B. Following *SNHG1* knockdown, the degree of caspase 3 cleavage decreased in response to docetaxel, with 0.58 and 0.21 as much observed than in siCTRL PC3 and C4-2B, respectively (Fig. 7). We obtained similar results in DU-145 (Additional file 3), and observed reduced PARP1 cleavage in C4-2B and DU-145 (Additional files 3 and 4) Thus, apoptosis is decreased in docetaxel treated cells deficient in *SNHG1*.

These data show that eliminating the activity of *SNHG1* protects cells from the cell cycle effects of docetaxel, and may reduce cytotoxicity. When *SNHG1* is present, cells treated with docetaxel display a G_2_ arrest and increased apoptosis. When *SNHG1* levels are suppressed by RNAi, the G_2_ arrest is eliminated and apoptosis is decreased.

### Correlation between *SNHG1* levels and docetaxel sensitivity

**Fig. 8:**
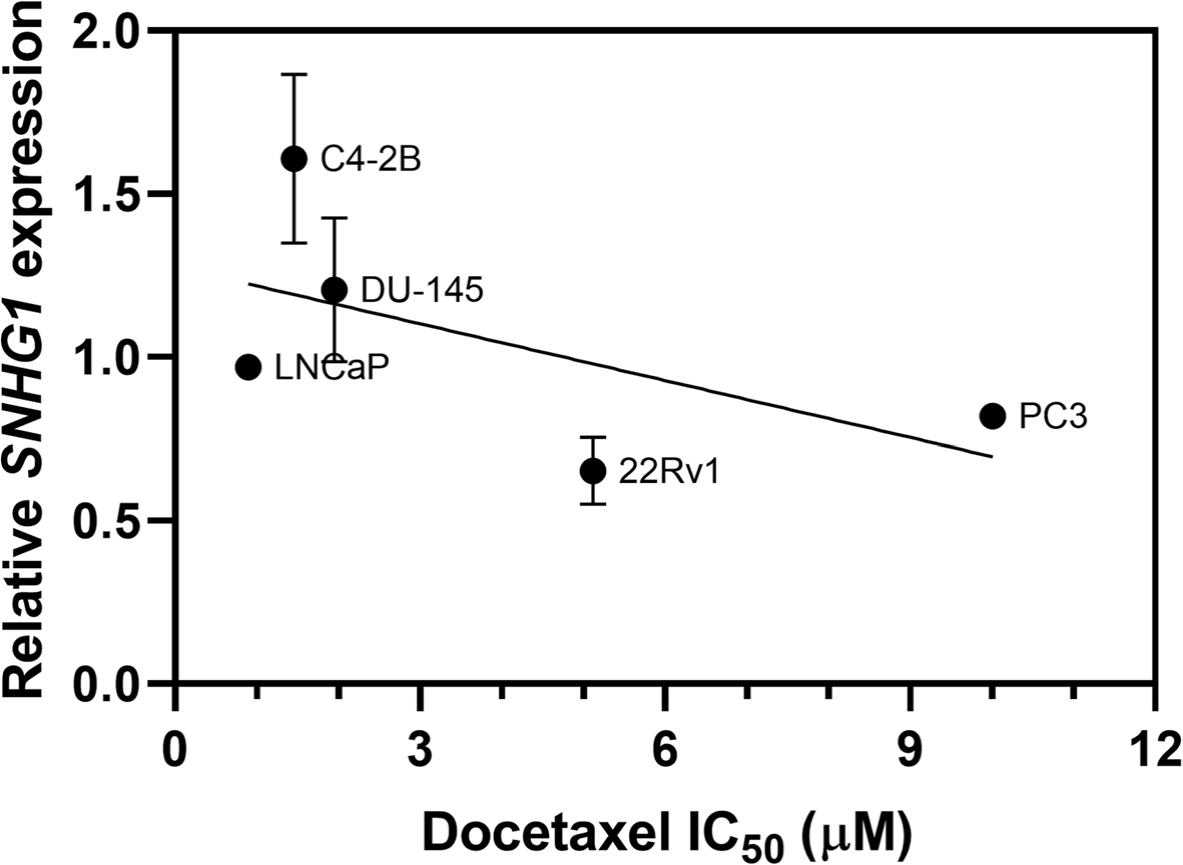
Correlation between *SNHG1* expression and sensitivity to docetaxel. SNHG1 levels were measured in various prostate cancer cell lines by RT-qPCR and correlated to docetaxel sensitivity as determined by an MTS assay

We determined endogenous *SNHG1* levels in a panel of prostate cancer cell lines and correlated expression to measured docetaxel IC_50_ values (Fig. 8). We found *SNHG1* levels between the lowest (22Rv1) and highest (C4-2B) expressing cell line to vary by as much as 2.5-fold. There was a small, but statistically significant, inverse correlation between *SNHG1* expression and docetaxel IC_50_ (*P* = 0.035; Deming model II regression). This supports the hypothesis that increased *SNHG1* expression leads to increased sensitivity to docetaxel.

## Discussion

Here we observe and characterize the biological effects of *SNHG1* on prostate cancer cells through selectively suppressing its expression using RNAi. The data show that *SNHG1* is involved in cell cycle and proliferation, partly through blocking G_0_ entry. When *SNHG1* is suppressed, even incompletely, cells reduce or cease proliferation, reduce or cease DNA synthesis, and accumulate in G_0_ phase. Meanwhile, cell cycle distribution in other phases (S, G_2_/M) is largely unchanged in C4-2B. Cell cycle distribution cannot be quantified by PI in PC3 cells due to polyploidy, but our supplemental data with DU-145 and LNCaP cells show similar results to C4-2B after *SNHG1* silencing. Interestingly, silencing *SNHG1* in benign prostate cancer cells (PNT2) has just a minor impact on DNA synthesis.

*SNHG1* expression varies with cell cycle under normal culture conditions. The quiescent (G_0_) population expresses lower levels of *SNHG1* than the G_1_ or S/G_2_/M populations, suggesting *SNHG1* has a function in cycling cancer cells. Quiescent and slowly dividing cells are resistant to chemotherapy [25, 26]. *SNHG1*-deficient cells treated with docetaxel showed decreased apoptosis and absence of the distinctive G_2_ arrest that normally occurs after treatment with this drug, indicating resistance. Remarkably, these effects are observed under RNAi conditions that result in a roughly 60% *SNHG1* knockdown. Furthermore, there is a small, but significant negative correlation between *SNHG1* expression and docetaxel IC_50_ in a panel of prostate cancer cell lines, which is consistent with our data indicating reduced apoptosis after *SNHG1* knockdown.

Our data is supportive of previous limited findings. You et al. [10] showed knockdown of *SNHG1* results in fewer colonies in a colony assay, suggesting a role in colony formation that could be attributed to cell cycle. Wan et al*.* analyzed prostate cancer in the TCGA database to find results similar to ours [6]. High *SNHG1* expression was associated with higher pathological stage and Gleason score, with a significantly reduced time to biochemical recurrence. These authors also found *SNHG1* to be androgen responsive- levels decreased after treatment with dihydrotestosterone, and increased when the androgen receptor was silenced by RNAi. However, these authors also found *SNHG1* levels to *increase* after dyhydrotestosterone treatment in a microarray experiment. Further investigation of this is clearly warranted, given the importance of the androgen receptor in prostate cancer progression and treatment, and that *SNHG1* levels dramatically affect quiescence and cell cycle as we have shown here.

These activities we describe for *SNHG1* have not previously been legitimately characterized in detail. The majority of literature on *SNHG1* focuses on proposed sponging activity, where *SNHG1* binds various miRNAs to affect specific gene expression. However, the literature lists interactions with at least 72 unique miRNAs, which are proposed to regulate at least 60 unique mRNAs. Many of these publications have been reported to have concerning data [27], or have been retracted, including the first report of *SNHG1* sponging activity in 2016. To our knowledge, none of the *SNHG1*-miRNA-mRNA interactions have been confirmed by a subsequent study by any group. This body of literature follows a consistent template which we suspect to be the result of commercialized production of fictional papers for profit (paper mills) as discussed by Else et al. and J. Christopher [14, 15].

*SNHG1* may exert its activity through direct RNA–protein interactions. Yang et al. used an RNA pulldown assay followed by mass spectrometry to identify proteins interacting with *SNHG1* in neuroblastoma cells [12]. One protein, matrin-3, was validated by a RIP (RNA immunoprecipitation) assay using anti-matrin-3 antibody. Matrin-3 is involved in RNA splicing and processing, suggesting a role for *SNHG1* in this process. *SNHG1* was also found to interact with PP2A-c to promote bladder cancer invasion [13]. *SNHG1* inhibited the interaction between PP2A-c and c-Jun to promote c-Jun phosphorylation and increased expression of MMP2, a protein important for cell invasion. *SNHG1* also promoted autophagy, resulting in increased *miR-34a* instability. *MiR-34a* is a miRNA which binds the 3’ UTR of *MMP2* mRNA. This resulted in less miRNA-mediated inhibition of *MMP2* mRNA, and therefore increased MMP2 protein. Notably, there was no demonstration of direct *SNHG1* binding to *miR-34a*, suggesting an indirect mechanism possibly related to the autophagy process.

Prostate cancer has a slow clinical course, tumors contain few proliferating (Ki67^+^) cells, and the disease can become dormant for decades, followed by reactivation and aggressive growth [1, 2]. The mechanism of clinical dormancy, and signals that reactivate tumors, are not thoroughly understood. Cells in G_0_ phase are considered quiescent, but have the ability to re-enter the cell cycle. Clinical dormancy may be the result of cells entering G_0_ until stimulated to restart the cell cycle, leading to recurrence. External signals from the bone microenvironment, a common site of prostate cancer metastasis, play a role in quiescence. Bone secretes factors such as TGFβ2, GDF10, and others, which induce cellular quiescence and dormancy in some prostate cancer cell lines [28, 29]. Wnt5a from the bone microenvironment also induces prostate cancer dormancy [30]. Elucidation of mechanisms promoting quiescence or re-activation, may give insight into how cells remain inactive for extended periods.

Other non-coding RNAs have been shown to affect quiescence and cell cycle through diverse mechanisms. In pancreatic cancer, the lncRNA *GAS5* regulates quiescence in cancer stem cells, which by extension, affects tumor recurrence [4]. LncRNAs have been shown to be involved in trimethylation of histone H4 at lysine 20, a process associated with quiescence [31]. Our study links *SNHG1* to quiescence, but the molecular basis for this activity is currently under investigation.

## Conclusions

We show that *SNHG1* plays a role in cell proliferation such that loss of *SNHG1* blocks cell exit from G_0_, leading to an accumulation of quiescent cells and a cessation of cell expansion in culture. Furthermore, *SNHG1* expression correlates with the quiescent versus cycling state of unmanipulated prostate cancer cells. DNA synthesis in prostate cancer, but not benign, cells virtually ceases in the absence of *SNHG1*. *SNHG1*-deficient cells resist G_2_ arrest when exposed to docetaxel, and the data suggest a decrease in apoptosis, indicating *SNHG1* levels influence docetaxel resistance. Elucidation of the molecular mechanism of these effects is important because as we have shown, *SNHG1* levels correlate with prostate cancer metastasis and impact clinical outcome. Then, targeting activities *SNHG1* is involved in could play a role in prostate cancer treatment, and the biology of tumor dormancy and recurrence.

## Supplementary Information

**Additional file 1.** Knockdownof *SNHG1*reduces DNA synthesis in LNCaPand DU-145 cells. (A) Assessmentof *SNHG1*knockdown in LNCaPand DU-145 cells. (B) Flow cytometryplot of EdUlabeled cells with or without *SNHG1*knockdown. (C) Quantitationof the proportion of EdU+cells after transfection with siCTRLor siSNHG1.All graphs depict mean±SD, *N*=3. Statistical analysis done using Studentt test: *P<0.05; **, *P*<0.01.

**Additional file 2.** *SNHG1*silencing is protective against DTX in LNCaPandDU-145 cells. (A) Representative histograms of PI stained LNCaPandDU-145 with or without *SNHG1*knockdown and with or without treatment with20 nMDTX. (B) Results of quantitation of apoptotic, G0/G1,S, and G2/Mpopulations in untreated or DTX treated, *SNHG1*silenced or not, cells.Data represent mean ±SD, *N*=4. Statistical analysis was done usingStudent’s t test: ns, not significant; *P<0.05; **, *P*<0.01.

**Additional file 3.** *SNHG1*silencing results in reduced G2phaseand apoptosis markers in DU-145 cells after DTX treatment. Western blotsshowing the apoptosis marker, cleaved caspase 3 and cleaved PARP1, and the G2marker,cyclin B1. β-actin is loading control. In (A), displayed is the sameblot reprobedfor different proteins. In (B), displayed are differentexposures of the same blot probed for caspase 3 (full length and cleavagefragment) and β-actin. Numbers indicate band density versus siCTRL, normalizedto β-actin.

**Additional file 4.** Original,full-length images from Western blotting, corresponding to cropped images shownin Fig. 7. *SNHG1*silencing results in reduced G2phaseand apoptosis markers after DTX treatment. Western blots showing the apoptosismarkers, cleaved caspase 3 and cleaved PARP1, and the G2marker,cyclin B1. β-actin is loading control. For PC3 (A), two separate blotsare shown. For C4-2B (B), a single blot was probed for multipleproteins. Numbers indicate band density versus siCTRL, normalized to β-actin.

## Data Availability

The datasets used and/or analysed during the current study are available from the corresponding author on reasonable request. Clinical data is available from the cBioPortal repository (https://www.cbioportal.org/), specifically, the TCGA, PanCancer Atlas (https://www.cbioportal.org/study/summary?id=prad_tcga_pan_can_atlas_2018) and MSK, Cancer Cell 2010 (https://www.cbioportal.org/study/summary?id=prad_mskcc) datasets.

## Abbreviations

DTX: Docetaxel
EdU: 5-Ethynyl-2’-deoxyuridine
HR: Hazard ratio
lncRNA: Long non-coding RNA
PI: Propidium iodide
RIP: RNA immunoprecipitation
TCGA: The cancer genome atlas
