# Supplementary material for: SNHG1 opposes quiescence and promotes docetaxel sensitivity in prostate cancer
Source: BMC Cancer. 2023 Jul 18;23:672. doi: 10.1186/s12885-023-11006-x (PMC10353248; doi:10.1186/s12885-023-11006-x)
Supplement: Supplementary file 1 — Additional file 1. Knockdownof SNHG1reduces DNA synthesis in LNCaPand DU-145 cells. (A) Assessmentof SNHG1knockdown in LNCaPand DU-145 cells. (B) Flow cytometryplot of EdUlabeled cells with or without SNHG1knockdown. (C) Quantitationof the proportion of EdU+cells after transfection with siCTRLor siSNHG1.All graphs depict mean±SD, N=3. Statistical analysis done using Studentt test: *P<0.05; **, P<0.01. [file 12885_2023_11006_MOESM1_ESM.pdf]

Additional File 1

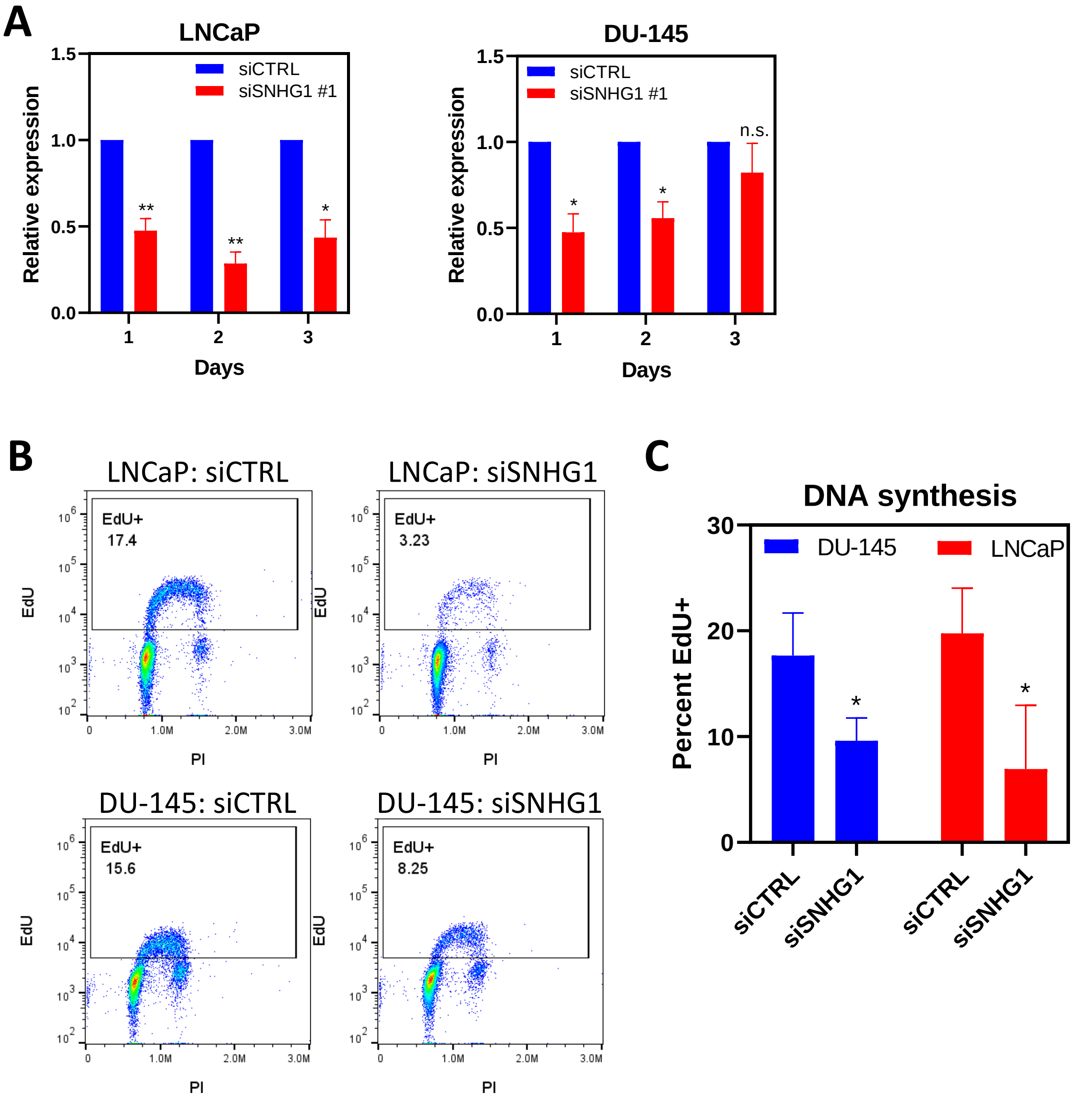

Additional File 1. Knockdown of *SNHG1* reduces DNA synthesis in LNCaP and DU-145 cells. **(A)** Assessment of *SNHG1* knockdown in LNCaP and DU-145 cells. **(B)** Flow cytometry plot of EdU labeled cells with or without *SNHG1* knockdown. **(C)** Quantitation of the proportion of EdU^+ cells after transfection with siCTRL or siSNHG1. All graphs depict mean ± SD, *N*=3. Statistical analysis done using Student t test: *, P<0.05; **, P<0.01.
