# Supplementary material for: SNHG1 opposes quiescence and promotes docetaxel sensitivity in prostate cancer
Source: BMC Cancer. 2023 Jul 18;23:672. doi: 10.1186/s12885-023-11006-x (PMC10353248; doi:10.1186/s12885-023-11006-x)
Supplement: Supplementary file 2 — Additional file 2. SNHG1silencing is protective against DTX in LNCaPandDU-145 cells. (A) Representative histograms of PI stained LNCaPandDU-145 with or without SNHG1knockdown and with or without treatment with20 nMDTX. (B) Results of quantitation of apoptotic, G0/G1,S, and G2/Mpopulations in untreated or DTX treated, SNHG1silenced or not, cells.Data represent mean ±SD, N=4. Statistical analysis was done usingStudent’s t test: ns, not significant; *P<0.05; **, P<0.01. [file 12885_2023_11006_MOESM2_ESM.pdf]

Additional File 2

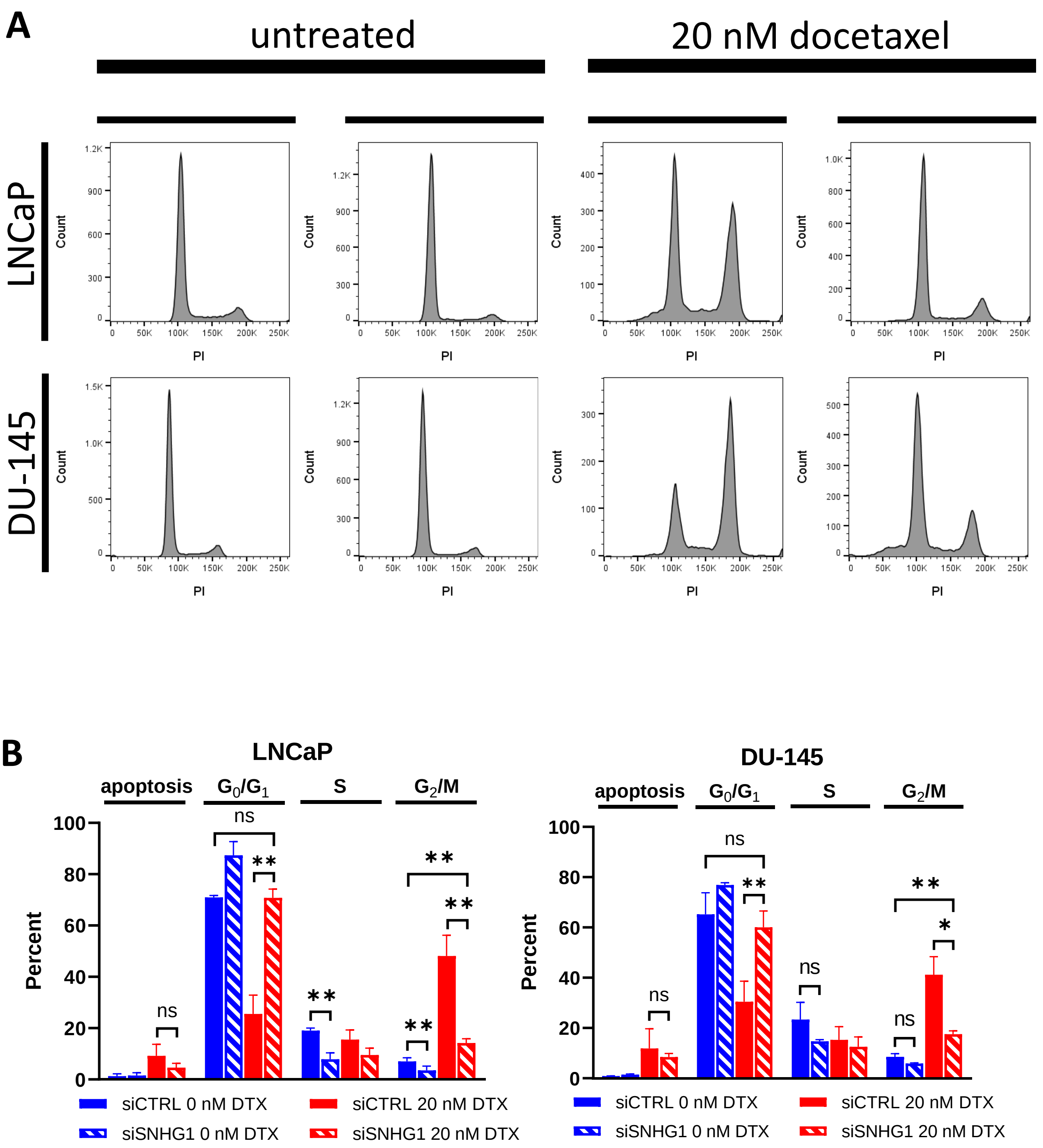

Additional File 2. *SNHG1* silencing is protective against DTX in LNCaP and DU-145 cells. **(A)** Representative histograms of PI stained LNCaP and DU-145 with or without *SNHG1* knockdown and with or without treatment with 20 nM DTX. **(B)** Results of quantitation of apoptotic, G_0/G_1, S, and G_2/M populations in untreated or DTX treated, *SNHG1* silenced or not, cells. Data represent mean ± SD, *N*=4. Statistical analysis was done using Student's t test: ns, not significant; *, P<0.05; **, P<0.01.
