# Supplementary material for: SNHG1 opposes quiescence and promotes docetaxel sensitivity in prostate cancer
Source: BMC Cancer. 2023 Jul 18;23:672. doi: 10.1186/s12885-023-11006-x (PMC10353248; doi:10.1186/s12885-023-11006-x)
Supplement: Supplementary file 3 — Additional file 3. SNHG1silencing results in reduced G2phaseand apoptosis markers in DU-145 cells after DTX treatment. Western blotsshowing the apoptosis marker, cleaved caspase 3 and cleaved PARP1, and the G2marker,cyclin B1. β-actin is loading control. In (A), displayed is the sameblot reprobedfor different proteins. In (B), displayed are differentexposures of the same blot probed for caspase 3 (full length and cleavagefragment) and β-actin. Numbers indicate band density versus siCTRL, normalizedto β-actin. [file 12885_2023_11006_MOESM3_ESM.pdf]

Additional File 3

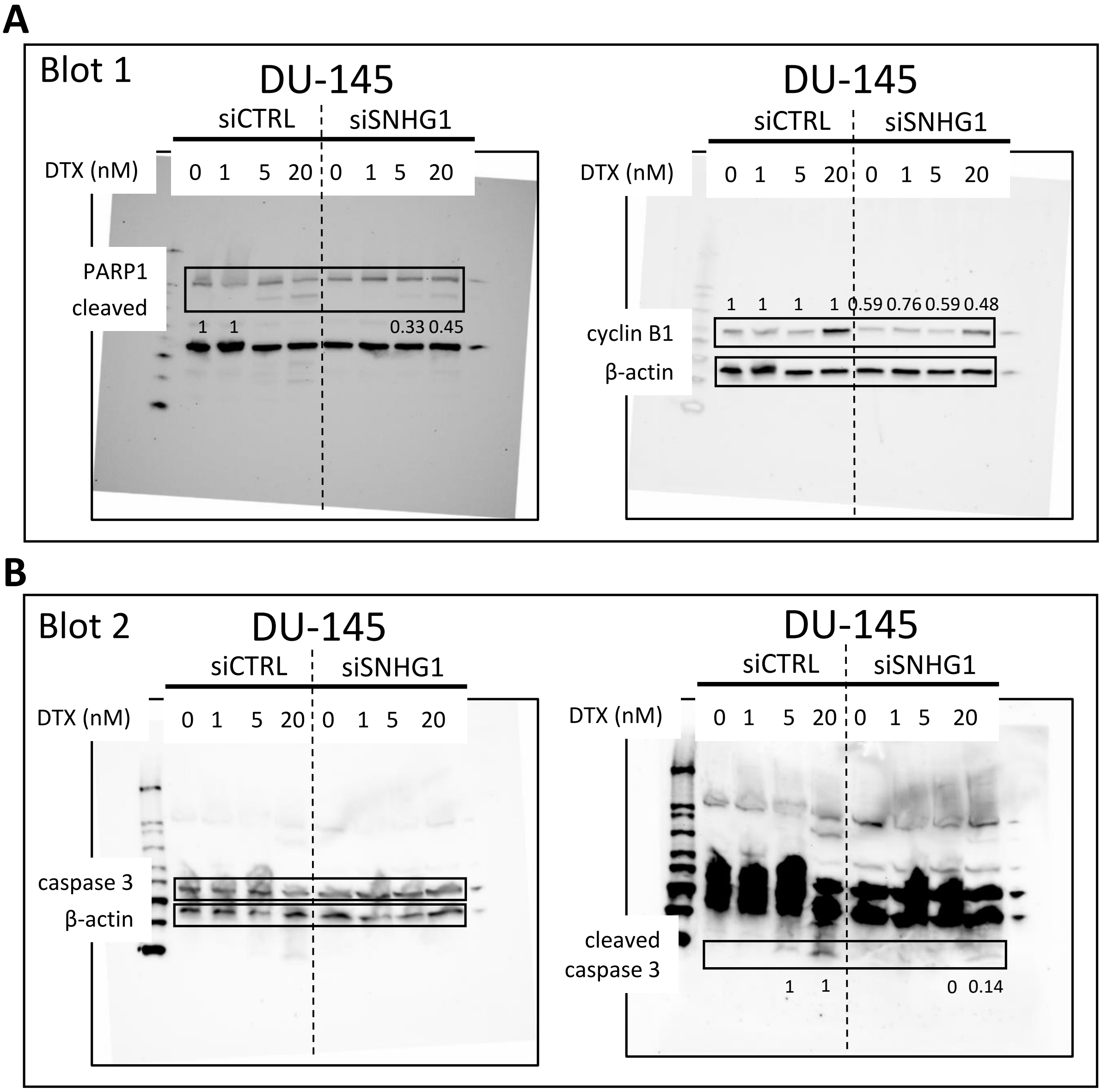

Additional File 3. *SNHG1* silencing results in reduced G_2 phase and apoptosis markers in DU-145 cells after DTX treatment. Western blots showing the apoptosis marker, cleaved caspase 3 and cleaved PARP1, and the G_2 marker, cyclin B1. β-actin is loading control. In **(A)**, displayed is the same blot reprobed for different proteins. In **(B)**, displayed are different exposures of the same blot probed for caspase 3 (full length and cleavage fragment) and β-actin. Numbers indicate band density versus siCTRL, normalized to β-actin.
