# Supplementary material for: SNHG1 opposes quiescence and promotes docetaxel sensitivity in prostate cancer
Source: BMC Cancer. 2023 Jul 18;23:672. doi: 10.1186/s12885-023-11006-x (PMC10353248; doi:10.1186/s12885-023-11006-x)
Supplement: Supplementary file 4 — Additional file 4. Original,full-length images from Western blotting, corresponding to cropped images shownin Fig. 7. SNHG1silencing results in reduced G2phaseand apoptosis markers after DTX treatment. Western blots showing the apoptosismarkers, cleaved caspase 3 and cleaved PARP1, and the G2marker,cyclin B1. β-actin is loading control. For PC3 (A), two separate blotsare shown. For C4-2B (B), a single blot was probed for multipleproteins. Numbers indicate band density versus siCTRL, normalized to β-actin. [file 12885_2023_11006_MOESM4_ESM.pdf]

Additional File 4

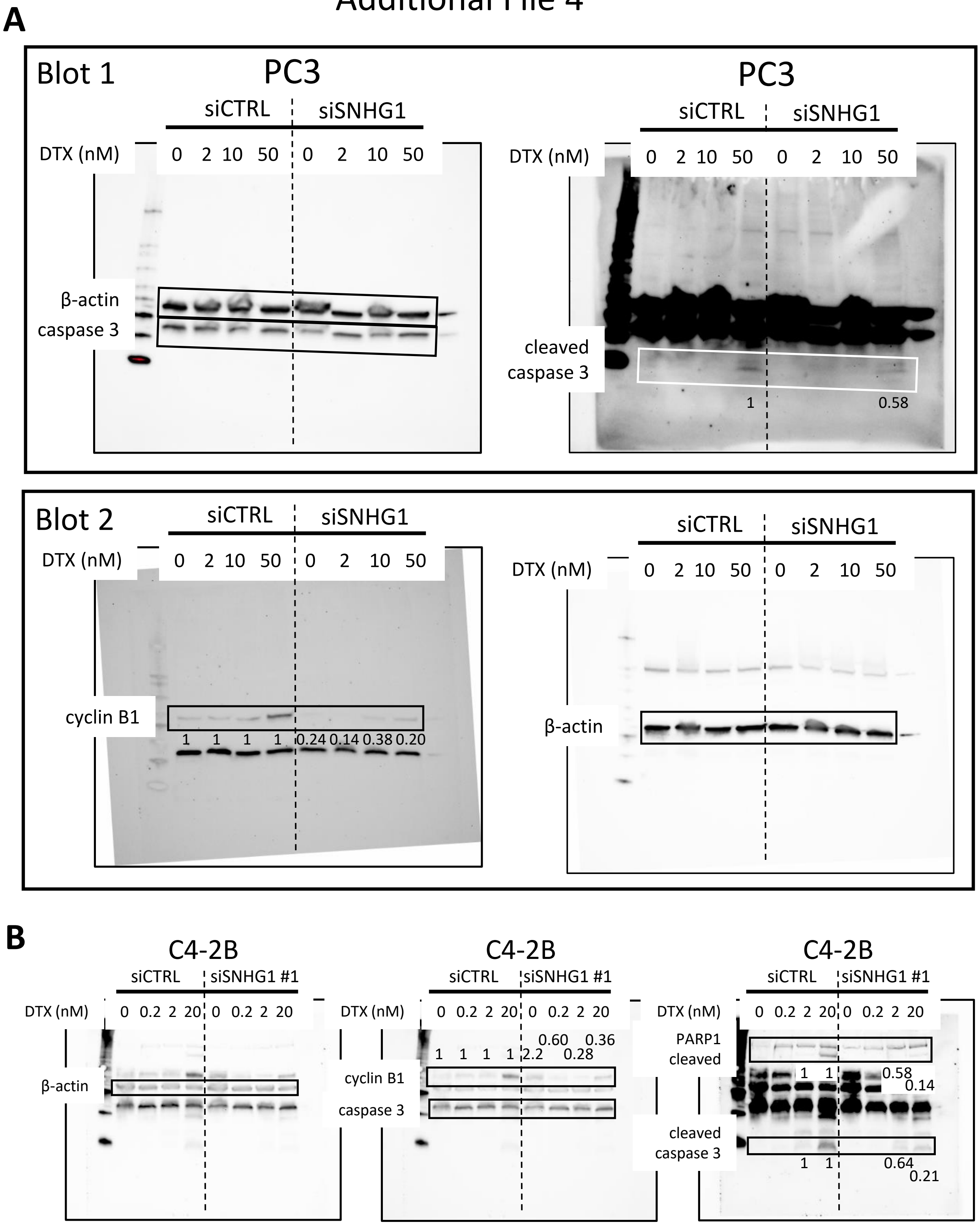

Additional File 4. Original, full-length images from Western blotting, corresponding to cropped images shown in Fig. 7. *SNHG1* silencing results in reduced G_2 phase and apoptosis markers after DTX treatment. Western blots showing the apoptosis markers, cleaved caspase 3 and cleaved PARP1, and the G_2 marker, cyclin B1. β-actin is loading control. For PC3 **(A)**, two separate blots are shown. For C4-2B **(B)**, a single blot was probed for multiple proteins. Numbers indicate band density versus siCTRL, normalized to β-actin.
